# A study of genetic variants associated with skin traits in the Vietnamese population

**DOI:** 10.1186/s12864-023-09932-y

**Published:** 2024-01-11

**Authors:** Tham Hong Hoang, Duc Minh Vu, Giang Minh Vu, Thien Khac Nguyen, Nguyet Minh Do, Vinh Chi Duong, Thang Luong Pham, Mai Hoang Tran, Ly Thi Khanh Nguyen, Han Thi Tuong Han, Thu-Thuy Can, Thai Hong Pham, Tho Duc Pham, Thanh Hong Nguyen, Huy Phuoc Do, Nam S. Vo, Xuan-Hung Nguyen

**Affiliations:** 1 GeneStory JSC, Hanoi, Vietnam; 2 Center for Biomedical Informatics, Vingroup Big Data Institute, Hanoi, Vietnam; 3 Hi-Tech Center and Vinmec-VinUni Institute of Immunology, Vinmec Healthcare System, Hanoi, Vietnam; 4 College of Health Sciences, VinUniversity, Hanoi, Vietnam; 5 View Plastic Surgery Center, Vinmec, Hanoi, Vietnam; 6 Vinmec Times City International Hospital, Vinmec Healthcare System, Hanoi, Vietnam

**Keywords:** Skin health, Genomic variants, Vietnamese cohort, Candidate genes, Skin-related traits

## Abstract

**Background:**

Most skin-related traits have been studied in Caucasian genetic backgrounds. A comprehensive study on skin-associated genetic effects on underrepresented populations such as Vietnam is needed to fill the gaps in the field.

**Objectives:**

We aimed to develop a computational pipeline to predict the effect of genetic factors on skin traits using public data (GWAS catalogs and whole-genome sequencing (WGS) data from the 1000 Genomes Project-1KGP) and in-house Vietnamese data (WGS and genotyping by SNP array). Also, we compared the genetic predispositions of 25 skin-related traits of Vietnamese population to others to acquire population-specific insights regarding skin health.

**Methods:**

Vietnamese cohorts of whole-genome sequencing (WGS) of 1008 healthy individuals for the reference and 96 genotyping samples (which do not have any skin cutaneous issues) by Infinium Asian Screening Array-24 v1.0 BeadChip were employed to predict skin-associated genetic variants of 25 skin-related and micronutrient requirement traits in population analysis and correlation analysis. Simultaneously, we compared the landscape of cutaneous issues of Vietnamese people with other populations by assessing their genetic profiles.

**Results:**

The skin-related genetic profile of Vietnamese cohorts was similar at most to East Asian cohorts (JPT: Fst = 0.036, CHB: Fst = 0.031, CHS: Fst = 0.027, CDX: Fst = 0.025) in the population study. In addition, we identified pairs of skin traits at high risk of frequent co-occurrence (such as skin aging and wrinkles (r = 0.45, *p* = 1.50e-5) or collagen degradation and moisturizing (r = 0.35, *p* = 1.1e-3)).

**Conclusion:**

This is the first investigation in Vietnam to explore genetic variants of facial skin. These findings could improve inadequate skin-related genetic diversity in the currently published database.

**Supplementary Information:**

The online version contains supplementary material available at 10.1186/s12864-023-09932-y.

## Introduction

As the largest organ in the human body, the skin acts as a barrier to regulate body temperature and prevent detrimental impacts from the external environment. Skin disorder is a common human health issue that severely weakens skin functions and hampers attractiveness [1]. Therefore, dermatological conditions are of immense concern for some individuals, especially females. As a result, there are increasing efforts to tackle these issues and improve skin health. One of the approaches is to study genetic factors affecting skin functions by providing an in-depth understanding the genetic factors needed to construct proper prevention and treatment strategies.

Many exogenous factors, such as overall health, lifestyle, daily diets, and other environmental factors, could influence skin conditions. Micronutrient deficiencies could also be a potential causative factor leading to cutaneous abnormalities. The most well-documented cutaneous condition-related micronutrients are B, C, and several fat-soluble vitamins, such as A, E, D, and K [2]. Moreover, several studies have confirmed the essential roles of genetics in developing some skin-related phenotypes and the association of various genetic variants with skin pigmentation, freckles, hairiness, and excessive sweating in certain populations [3–7]. Recently, the advent of the Human Genome Project, genome-wide association studies (GWAS), and next-generation sequencing (NGS) have enabled experts to identify candidate loci in the genes underlying skin-related traits and skin disorders. The act of investigating the frequency and annotations of genetic variants on these loci in populations may shed light on specific skin disorders for each country as well as the difference in specific skin-related issues between populations.

Even though Asian populations make up over 40% of the global population and comprise a significant genetic diversity, they are still underrepresented in current genomic studies. To date, skin genetics have been studied primarily in populations with European ancestry. The first genome-wide association study (GWAS) of skin pigmentation included 1,043 individuals from 51 European ancestry populations who identified major loci and specific polymorphisms affecting human skin color [7]. In 2018, a UK biobank-based GWAS was conducted to analyze a broad set of 23 highly heritable traits, including skin and vitamin D levels, tanning, and sunburn [5]. Aside from Europeans, several studies have been performed on Asian populations, such as Han Chinese, Indian, Japanese, and Korean populations [3, 4, 6, 8]. A large-scale GWAS of various skin phenotypes identified several novel skin-spot trait-associated signals neighboring *AKAP1/MSI2, BNC2, HSPA12A, PPARGC1B, and RAB11FIP2* and double-edged eyelid-related signals around *EMX2* in a Japanese female population [3]. In Korea, a GWAS analysis in 17,019 women revealed several genomic loci significantly associated with facial pigmented spots (e.g., *BNC2, PPARGC1B, MC1R, MFSD12*) [6]. These insights helped dermatologic researchers better understand the genetic underpinnings of skin-related phenotypic variation in Asian populations.

There are several ways to estimate an individual’s genetic contribution to a phenotype. The two most popular methods are the polygenic risk score (PRS) [9] and GRS-RAC [10] (also called the Count SNP Score or Top SNP Score). PRS estimates how the collection of one’s variants affects their risk for certain traits or diseases by using DNA information and data derived from large-scale genomic studies [9]. In contrast, the Top SNP Score only considers the genomic variants significantly associated with phenotypes. Therefore, the Top SNP Score is much simpler than PRS but requires tremendous work for database curation [10]. However, both of these approaches allow us to calculate the health condition-associated risks to guide healthcare decisions.

In the present study, we aimed to explore the unique pattern of cutaneous problems in Vietnam by investigating the correlation among genetic and nongenetic factors (collected by customized questionnaire) associated with various traits, including skin-related and micronutrient requirements. In addition, we compared the landscape of cutaneous issues based on associated variant profiles in Vietnamese people with other populations worldwide to explain the interpopulation variability in dermatological problems and provide an understanding to improve dermatological health.

## Materials and methods

### Study cohorts

i) Microarray dataset: This study included 96 Vietnamese adults aged from 25 to 50 with no remarkable medical history related to skin. After being consulted with dermatologists about the process and the objectives of the research, the dermatological issues of participants were assessed with a dermatoscope by the trained dermatologists (the assessment results are presented in Table 1). Then, clinicians interpreted the results and diagnosed the condition of the participant’s skin. All participants completed a survey on other skin-related information. All participants provided consent to participate in this study using written informed consent forms. These samples were processed with the Infinium Asian Screening Array-24 v1.0 (ASA) BeadChip.
ii) 1000 Vietnamese Genomes (VN1K) dataset: We used whole-genome sequencing (WGS) data of 1008 unrelated individuals from the 1000 Vietnamese Genomes Project (VN1K) by Vingroup Big Data Institute to investigate population-specific skin-related risks. In the VN1K study, subjects provided informed consent, and the study was approved by the Vinmec International Hospital Institutional Review Board with number 543/2019/QĐ-VMEC. The characteristics of 1008 individuals can be found on the official website of VN1K at https://genome.vinbigdata.org/.
iii) 1000 genome project phase 3 (1KGP3) dataset: We also used WGS data of 2504 unrelated individuals of five superpopulations of African (AFR), Ad Mixed American (AMR), East Asian (EAS), European (EUR), and South Asian (SAS) for comparison with the VN1K population. This 1KGP3 dataset has its own consent form.

**Table 1:** Mean VISIA assessment results

Trait	Angle	Percentile	Feature count	Score
Spots	Left	73.88	110.2	28.03
	Right	75.95	108.3	26.95
	Front	69.21	145.15	32.21
Wrinkles	Left	40.04	103.9	28.108
	Right	42.23	107.4	27.377
	Front	50.844	29.37	11.536
Texture	Left	46.23	1603	11.883
	Right	43.3	1660	12.568
	Front	43.211	2550.67	49.097
Pores	Left	51.98	552.1	14.953
	Right	51.91	556.8	15.121
	Front	48.065	962.54	52.441
UV spots	Left	87.98	260.4	16.338
	Right	86.33	261	16.661
	Front	88.35	369.514	17.738
Brown spots	Left	63.17	89.17	17.005
	Right	63.56	85.1	16.805
	Front	64.92	120	17.39
Red areas	Left	66.81	12.62	11.416
	Right	65.25	12.73	11.616
	Front	70.178	15.22	10.823
Porphyrins	Left	41.4	1588	17.421
	Right	43.52	1564	17.104
	Front	45.98	2563.26	71.155

### DNA extraction, purification, and genotyping

DNA was extracted from saliva specimens and purified using the MagMAX DNA Multi-Sample Ultra 2.0 Kit (Thermo Fisher Scientific, USA) according to the manufacturer’s instructions. After the single-base extension, the ASA chip was processed and scanned by the Illumina iScan System. The ASA chip is designed with more than 660,000 variants for medical research and related disease studies in East Asian populations [11]. A total of 94/96 samples had a calling rate greater than 98%. The.IDAT files (the format obtained from the Iscan machine for the ASA chip) were converted to.vcf, yielding 656,891 variants. Of these, 623,886 variants were located on autosomes. The research team used Shapeit4 [12] and Minimac4 [13] software to add imputation points with the genomes of 1000 Vietnamese Genomes people, removing bad variants with impute R2 < 0.3. Some criteria for selecting samples and variants: (i) samples: proportion of missing variants < 0.05 excluding blood-related samples, (ii) variants: missing proportions of variants to total samples < 0.05, variants with *p* value < $$ {10}^{-6}$$ following the Hardy-Weinberg principle, and with MAF (minor allele frequency) MAF < 0.01 were eliminated.

### Evaluation of 25 common skin-related traits

Skin traits can be temporary or permanent and directly influence quality of life. There are many different types of skin abnormalities. We investigated the 25 most common features that were reported to be associated to genetic variants: freckles, tanning response, UV protection ability, skin aging, elasticity, antioxidant capacity, and skin-related micronutrient requirements. Some of these present relationships with others; one issue could be the cause or the consequence of another. Generally, they can be categorized into four main groups: pigmentation, dermatitis, nutrition, and aging (Supplementary file, Figure S1).

### GWAS and SNP dataset curation for analysis

GWAS and other genetic association studies for skin features were collected from the GWAS Catalog and PubMed. In the GWAS Catalog, the search strategy was based on the keywords and their synonyms for 25 skin-related traits (i.e., “Freckle,” “Tanning response,” “UV protection,” “Skin sensitivity,” “Inflammatory cytokines,” “Skin aging,” “Elasticity,” “Antioxidant,” “Collagen,” “Stretch mark,” “Wrinkle,” “Glycation,” “Moisturizing,” “Acne,” “Eyelid,” “Vitamin A need,” “Vitamin B2 need”, “Vitamin B6 need”, “Vitamin B9 need”, “Vitamin B12 need”, “Vitamin C need,” “Vitamin D need,” “Vitamin K need,” “Omega need”). For searching studies from the Pubmed platform, we also used the aforementioned search terms combined with others representing genetic association studies, such as “Genome-wide association studies,” “genetic association,” “genomic study,” or “GWAS.”

Selected studies must report information regarding genetic variations associated with skin-related traits. We will observe summary data for every SNP that significantly modulates the risk of skin traits. The data extraction form included genetic variation information (SNP, gene, location), participant information (quantity, ethnicity, mean age, gender proportion, etc.), affected skin traits, and statistical data (effect size (beta) and *p* value of the associations). Identified variants were compared with other sets of SNPs from several vendors on the market, such as OmeCare (U.S.A.) and BioEasy (Malaysia), to obtain high confidence in consensus SNPs. The list of identified SNPs overlapped with the Vietnamese Genetic Variation Database from VN1K to establish a comprehensive variant dataset related to dermatology.

### Questionnaire forms

The questionnaire was constructed to build holistic metadata of all participants. All 96 participants in the microarray dataset completed the questionnaire regarding demographics (gender, age), body mass index (BMI), lifestyle (stress, frequency of exercise, sunlight exposure levels), hypersensitivity history, alcohol use, smoke exposure (active/passive), medications, nutrition (sweet eating habits, fruit-eating habits), skin problems in family members, and information about skin care (approaches, intensity, skin satisfaction levels). The characteristics and information retrieved from the questionnaire form are summarized in Table 2.

**Table 2:** Characteristics of 96 participants involved in the microarray dataset

Characteristics	N = 96	Characteristics	N = 96
Age(years) – mean (± SD)	37.2 (± 11.5)	Atopic dermatitis – no. (%)	11 (11.5%)
Male/Female	28/68	Urticaria/Rashes – no. (%)	12 (12.5%)
Height		Exercise – no. (%)	55 (57.3%)
Male – mean (± SD)	169.8 (± 5.7)	Smoke exposure – no. (%)	14 (14.6%)
Female – mean (± SD)	157.2 (± 4.3)	Stay up late/insomnia/Stress – no. (%)	60 (62.5%)
Weight		Sweet eating habit – no. (%)	41 (42.7%)
Male – mean (± SD)	66.5 (± 10.2)	Fruit eating habit – no. (%)	77 (80.2%)
Female – mean (± SD)	53.9 (± 6.7)	Frequently alcohol used - no. (%)	25 (26.0%)
Skin care		Family members suffering from skin problems - no. (%)	52 (54.2%)
Cleansing – no. (%)	50 (52.1%)	Intensive care – no. (%)	24 (25.0%)
Facial washing with cleaning preparations – no. (%)	75 (78.1%)	Current medications used - no. (%)	17 (17.7%)
Exfoliation – no. (%)	44 (45.8%)	Hypersensitivities	
Torner – no. (%)	43 (44.8%)	Drug – no. (%)	9 (9.4%)
Serum – no. (%)	38 (39.6%)	Food – no. (%)	16 (16.7%)
Peptide – no. (%)	5 (5.2%)	Other – no. (%)	11 (11.4%)
Moisturizing – no. (%)	54 (56.3%)	Water liters/day	
Nourishing mask – no. (%)	37 (38.5%)	< 1 L – no. (%)	5 (5.2%)
Supplement – no. (%)16 (16.7%)Sunscreen – no. (%)	53 (55.2%)	1-1.5 L – no. (%)	16 (16.7%)
Face massage – no. (%)	23 (24.0%)	1.5-2 L – no. (%)	66 (68.8%)
Spa – no. (%)	15 (15.6%)	2–3 L – no. (%)	9 (9.4%)
Supplement – no. (%)	16 (16.7%)	Skin satisfaction – no. (%)	24 (25.0%)

### Allele frequency analysis

We also use Weir and Cokerham’s weighted Fst [14] to compare the genetic structure of the Vietnamese population with other populations. Fst is a parameter of F-statistics developed by Weir and Cokerham to summarize population structure based on the theorem by Wright [15].

### Construction of a skin feature landscape for the Vietnamese population in comparison with others

**Fig. 1:**
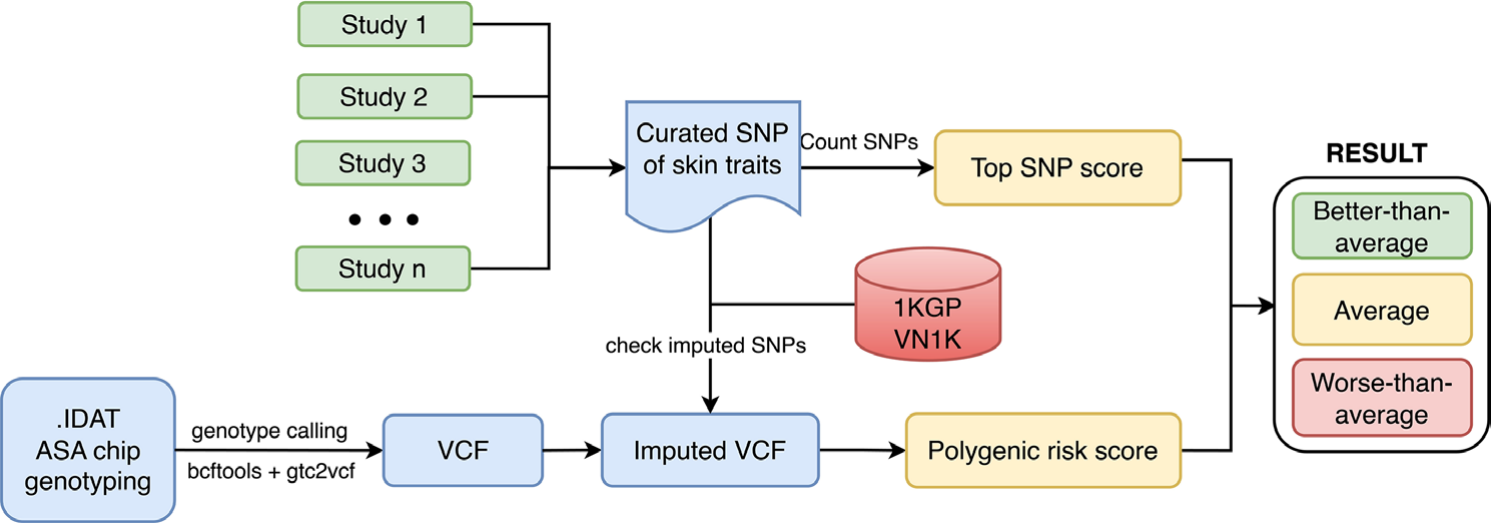
The process of SNP curation and division of VN1K by PRS and Top SNP methods into three equal intervals, equivalent to 3 statuses of “Better-than-average,” “Average,” and “Worse-than-average,” with increased risk of skin-related traits

To assess the genetic predisposition related to skin traits, we proposed the process of data curation and cohort categorization depicted in Fig. 1. If the GWAS sumstat data is available, the genetic score for skin traits should be calculated by PRS methods, otherwise, they could be estimated by GRS-RAC method.

We used the GRS-RAC method to assess individual genetic risk for skin traits for 96 participants in this study and 1008 Vietnamese people from VN1K [10]. Risk alleles (R) and nonrisk alleles (N) were counted at each locus. Genotype RR was counted as 2, RN as 1, and NN as 0. Then, the number of risk alleles was summed for each participant from the microarray dataset. In this study, this method was called “top SNP”.

To detect population-specific risks related to skin traits, we generated a landscape of skin conditions for Vietnamese and other populations derived from 1KGP3. Based on the range of skin-related SNPs, we divided the population into three intervals according to the quantity of variants carried (Top SNP score). Three intervals were named “better-than-average”, “average”, and “worse-than-average” skin conditions.

### Correlation analysis

The correlations of numerous factors included in the metadata and genetic risk of skin conditions of the microarray dataset, represented by their Top SNP score, were evaluated by Pearson correlation analysis to find their relationships. The levels of correlations ranged from − 1 to 1. The closer R is to 1, the more the correlated variable promotes the latter and vice versa. Specifically, 0.1≤$\left|R\right|$<0.3: weak correlation; 0.3≤$\left|R\right|$<0.5: moderate corelation; and $\left|R\right|$ ≥ 0.5: strong correlation. If R equals 0, there is no correlation between the two variables. A *p* value<0.05 indicated a significant correlation.

## Results

### Skin variation in Vietnamese populations compared with other populations

**Fig. 2:**
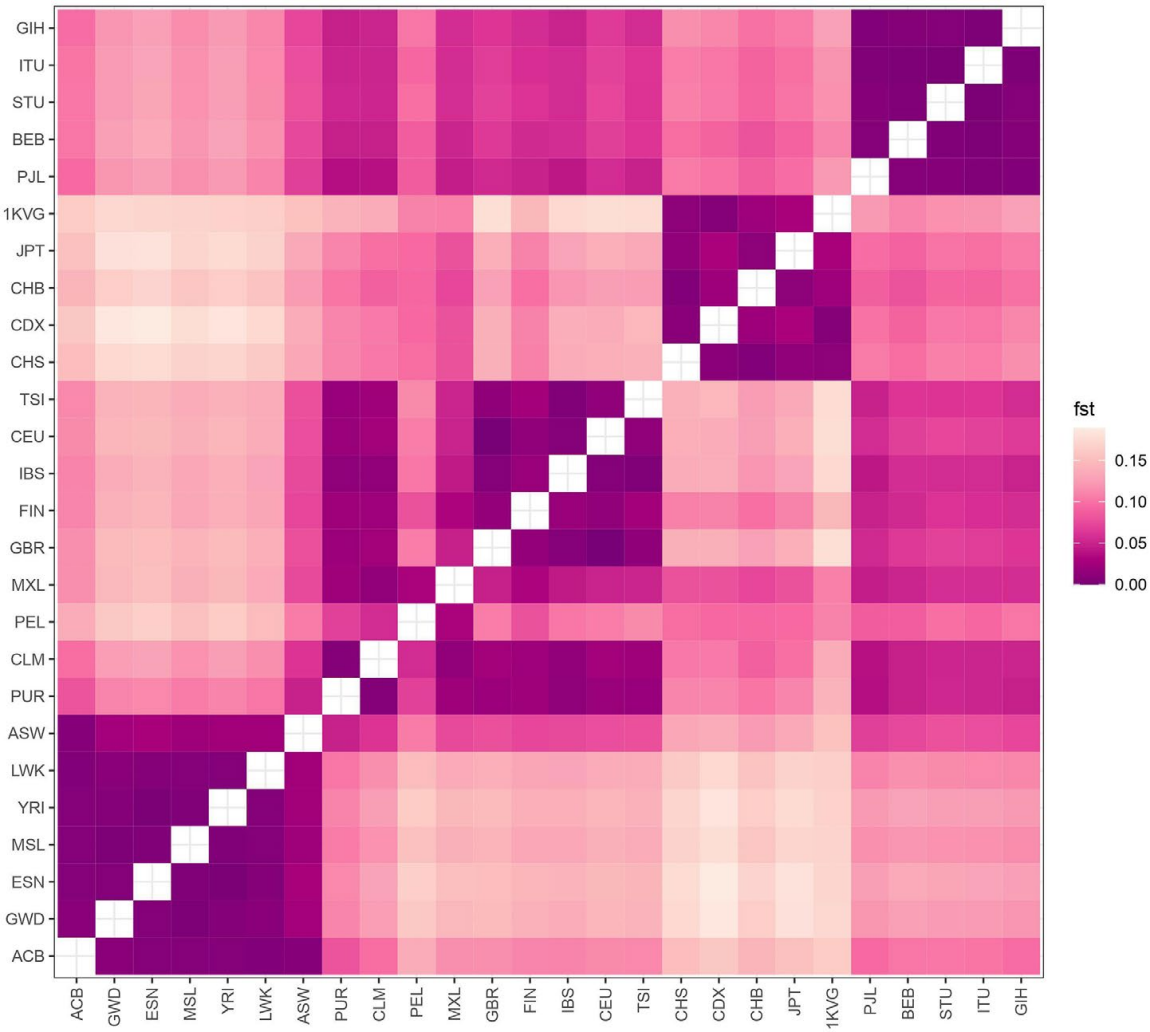
Heatmap displaying the Fst score for Vietnamese versus other populations from 1KGP3. 1000 Vietnamese genome project, East Asian (CHS: Han Chinese South; CDX: Chinese Dai in Xishuangbanna, China; CHB: Han Chinese Beijing, China; JPT: Japanese in Tokyo, Japan), South Asian (PJL: Punjabi in Lahore, Pakistan; BEB: Bengali in Bangladesh; STU: Sri Lankan Tamil in the U.K.; ITU: Indian Telugu in the U.K.; GIH: Gujarati Indians in Houston, Texas, USA); European (GBR: British From England and Scotland; FIN: Finnish in Finland; IBS: Iberian Populations in Spain; CEU: Utah residents (CEPH) with Northern and Western European ancestry; TSI: Toscani in Italia), American (PUR: Puerto Rican in Puerto Rico; CLM: Colombian in Medellín, Colombia; PEL: Peruvian in Lima Peru; MXL: Mexican Ancestry in Los Angeles CA USA), African (ACB: African Caribbean in Barbados; GWD: Gambian in Western Division - Mandinka; ESN: Esan in Nigeria; MSL: Mende in Sierra Leone; YRI: Yoruba in Ibadan, Nigeria; LWK: Luhya in Webuye, Kenya; ASW: African Ancestry in SW, USA

There is a noticeable distinction in allele frequency of variant-associated skin traits between different populations worldwide. We first analyzed the overall population distance using the average Weir and Cokerham’s weighted Fst for 85 aforementioned skin-related SNPs. The genetic profile associated with skin traits of the Vietnamese (VN1K) population was the most similar to that of the East Asian population. In particular, fixation index values (Fst) between Vietnam and these populations (JPT: Fst = 0.036, CHB: Fst = 0.031, CHS: Fst = 0.027, CDX: Fst = 0.025) are far less significant than those between Vietnam and South Asian (SAS), European (EUR), Ad Mixed American (AMR) or African (AFR) populations (Fst ≥ 0.09) (Fig. 2).

### The genomic landscape of skin traits of Vietnamese

**Fig. 3:**
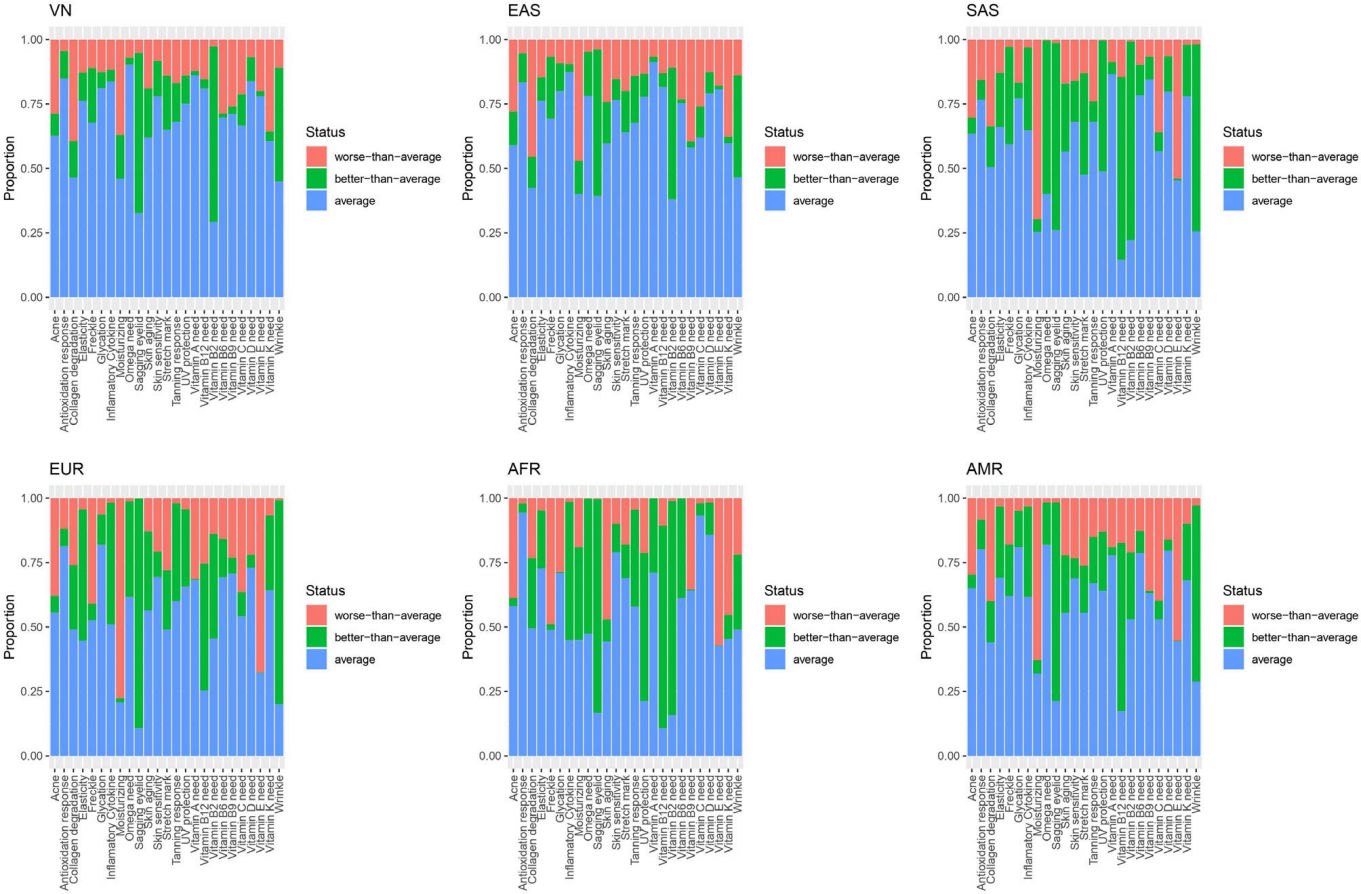
The genomic landscape of skin-related traits in the Vietnamese population and five superpopulations derived from the 1000 Genomes Project. VN: Vietnamese (VN1K); EAS: East Asian, AFR: African; AMR: American; EUR: European; SAS: South Asian

Figure 3 shows that the risks vary among different skin-related traits in Vietnamese people. For example, most Vietnamese people possess an advantageous genetic profile for antioxidant response. In other words, the majority of the Vietnamese population has a remarkable ability to defend their skin against oxidizing agents. In contrast, a higher proportion of the investigated population was shown to be at high risk of moisturizing, collagen degradation, and acne. These conditions rapidly worsen skin health and accelerate skin aging [16, 17]. Regarding skin-related micronutrient requirements, most Vietnamese people carry a favorable genotype for maintaining adequate serum levels of vitamin D and essential fatty acids (EFAs) (e.g., omega-3, omega-6); in contrast, they are likely to be at risk of suffering from deficiencies in vitamin C, vitamin B complex and vitamin K.

The genomic landscape of Vietnamese facial skin traits showed the highest similarity level with the East Asian population (EAS). In contrast, there are several differences between the Vietnamese people and other ancestries, such as European, African, and American (Table S1). Indeed, European and American genetic profiles have been shown to be more susceptible to freckles and moisturizing, whereas African people are at higher risk of wrinkles and glycation than Vietnamese people. Four superpopulations from 1KGP3 (except East Asian) are prone to vitamin E deficiency, while more than 75% of the Vietnamese population carries a benign genetic profile for this condition.

### Correlation among skin-related personal characteristics and top SNP scores

Based on the information retrieved from questionnaire forms and genotyping results of the microarray dataset, a correlation analysis was performed to identify potential relationships between several skin-related traits and genetic profiles (Table S2). As expected, we observed that in comparison between the genders, men had higher levels of alcohol consumption (r =-0.51, *p* = 6.18e-7), and men also exercised more frequently (r = -0.24, *p* = 0.026). In contrast, women often skincare and perform cosmetic interventions (r = 0.59, *p* = 5.03e-09; r = 0.30, *p* = 0.005, respectively); however, they had lower satisfaction with their skin condition (r = -0.28, *p* = 0.009). People tend to be interested in their skin health by age, which could be seen through the fact that older people skincare more carefully and frequently (r = 0.26, *p* = 0.019).

By sharing risk variants, or causality relationships, a few traits show a significant correlation (e.g., collagen degradation and elasticity (r = 0.84, *p* = 8.0e-4), skin aging and wrinkles (r = 0.45, *p* = 1.50e-5). Aside from these pairs, several skin traits could be present in the Vietnamese population, such as collagen degradation often appearing with moisturizing (r = 0.35, *p* = 1.1e-3), freckles and inflammatory cytokines (r = 0.23, *p* = 0.03). Moreover, some cutaneous issues tend to come with micronutrient deficiency. It could be seen at the pair of tanning responses and vitamin B9 level (r = -0.33, *p* = 2.6e-3) or moisturizing and vitamin A level (r = 0.26, *p* = 0.02). These findings could suggest their relationship and provide information for constructing the most proper strategy to improve skin health.

## Discussion

The advent of VN1K offers an excellent database for obtaining a better understanding of genomics and their role in various health problems. By using the genetic profile of all participants in VN1K, allele frequency analysis and phenotype landscape have shown a remarkable distinction between populations, suggesting that aside from environmental factors (e.g., climate, living habits, etc.) [18], the genetic profile is an important cause leading to population-specific facial skin traits. These findings could partly explain the different patterns of skin traits in populations. For example, Vietnamese people often deal with collagen degradation and acne, while the significant problems of Europeans are freckles and moisturizing. Therefore, it is crucial to understand the unique skin problems and their causes in each population to properly construct a specific strategy to defend against their high-risk issues.

We identified correlations between several pairs of skin-related traits, which suggested a tendency for their co-occurrences in the Vietnamese population. These phenomena could be attributed to the causal relationship or sharing the related variants between two traits (e.g., collagen degradation and elasticity, or skin aging and wrinkle); however, in cases of the remaining pairs (e.g., collagen degradation and moisturizing, moisturizing and vitamin A level, or freckle and inflammatory cytokine, etc.), they might suggest potential associations, particularly in Vietnamese individuals.

Most variants of interest in this study was explored in population with European descents. Specifically, 28 studies selected were carried out on Caucasians, while merely 13 studies were performed on population with Asian descents and the remaining 11 studies conducts with a sample of mixture descents. Therefore, it could pose a number of bias on the findings due to the difference in genetic background. It is suggested to validated the effect of these variants on Asian populations.

Aside from using 2 huge genomic datasets VN1K and 1KGP3 to explore the population-specific characteristic related to skin, we also used a pilot validation dataset including 96 healthy Vietnamese adults to validate these results and examine the ability of applying SNP microarray in the assessment of genetic predisposition. The modest sample size could induce several noise in computational analysis and also limit the statistic power of explored correlation. However, the findings from analysing the dataset can be the signal for later studies in this field.

## Conclusion

This is the first study on the Vietnamese population to investigate the genetic features that play an essential role in skin health. However, further functional characterizations of the investigated genes are warranted to elucidate their contribution to skin-related traits. By examining several skin-associated genetic variants in the Vietnamese population, this study could improve inadequate skin-related genetic diversity in the currently published database.

### Electronic supplementary material

Below is the link to the electronic supplementary material.

Supplementary Material 1

## Data Availability

96 ASA genotyping samples have been added into GEO dataset with the ID of GSE248483 including ASA genotyping raw files, processed files and metadata. Data from VN1K can be accessed at https://genome.vinbigdata.org after the registration.

## Code availability

Our wrapped pipeline for generating Polygenic Risk Score and all processed data is available for public use at https://github.com/vmgiang/skin-paper.

## Abbreviations

VN1K: 1000 Vietnamese Genomes Project
1KGP3: 1000 Genomes Project Phase 3
AFR: African
AMR: Ad Mixed American
ASA: Infinium Asian Screening Array-24 v1.0 BeadChip
EAS: East Asian
EFA: Essential fatty acid
EUR: European
GWAS: Genome-wide association studies
LD: Linkage disequilibrium
NGS: Next-generation sequencing
PRS: Polygenic Risk Score
SAS: South Asian
SNP: Single Nucleotide Polymorphism
WGS: Whole Genome Sequencing
