# Supplementary material for: A study of genetic variants associated with skin traits in the Vietnamese population
Source: BMC Genomics. 2024 Jan 11;25:52. doi: 10.1186/s12864-023-09932-y (PMC10785522; doi:10.1186/s12864-023-09932-y)
Supplement: Supplementary file 1 — Supplementary Material 1 [file 12864_2023_9932_MOESM1_ESM.docx]

**Supplementary file**


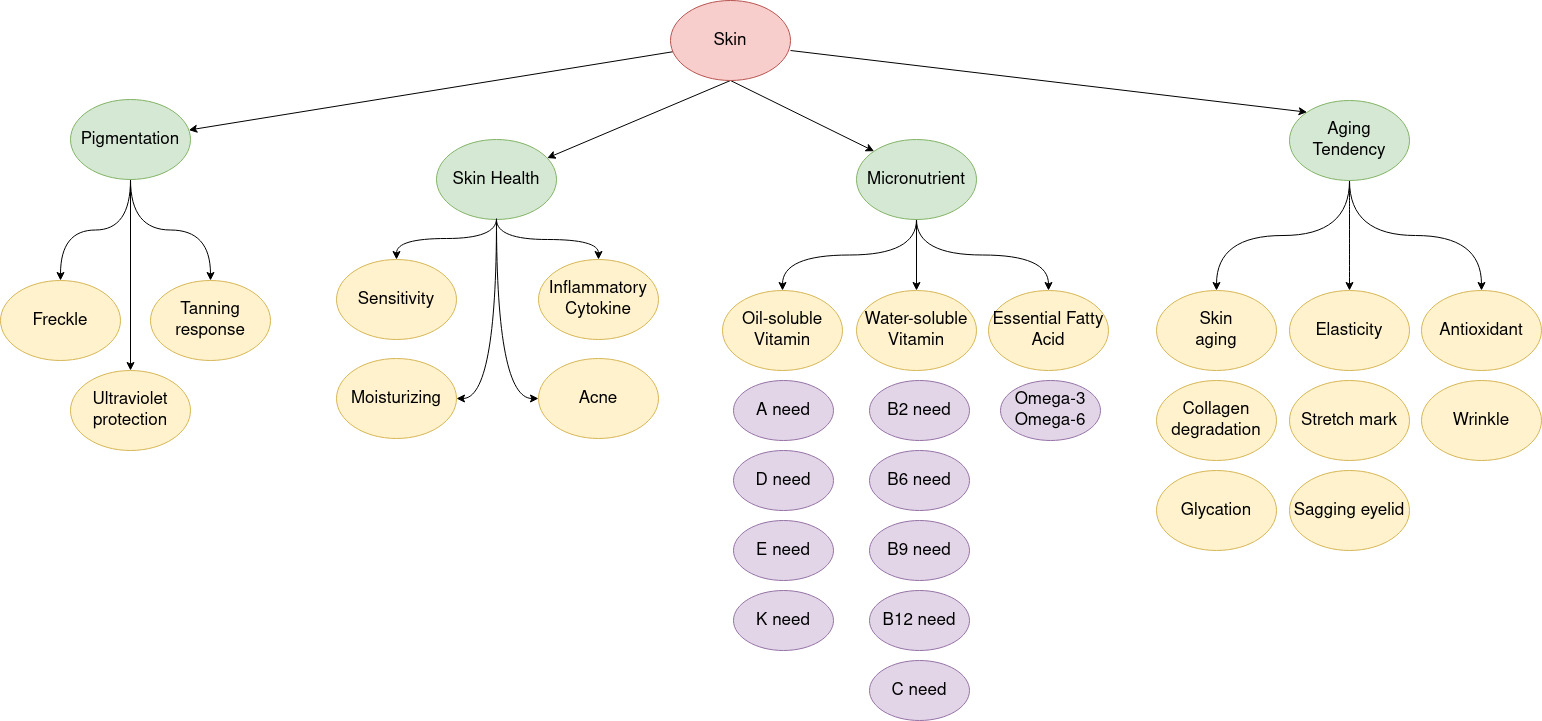


**Figure S1**. The structure 25 skin-related traits to illustrate relationships in four groups of Pigmentation, Dermatitis, Nutrition and Aging.

**Table S1.** The allele frequency of skin-related SNPs.

| **rsID** | **Associated traits** | **Reference** | **PubMed**  **ID** | **AF_VN1K** | **AF_96ASA** | **AF_1kGP** | **AF_AFR** | **AF_AMR** | **AF_EAS** | **AF_EUR** | **AF_SAS** |
| --- | --- | --- | --- | --- | --- | --- | --- | --- | --- | --- | --- |
| rs1801222 | Vitamin B12 concentration | John W 2015 | 25948668 | 0.846 | 0.880 | 0.799 | 0.813 | 0.797 | 0.842 | 0.705 | 0.836 |
| rs16927253 | Sagging eyelid | Vincent L 2018 | 29654602 | 0.212 | 0.188 | 0.120 | 0.066 | 0.127 | 0.239 | 0.053 | 0.150 |
| rs4746957 | Sagging eyelid | Vincent L 2018 | 29654602 | 0.216 | 0.188 | 0.125 | 0.083 | 0.127 | 0.239 | 0.053 | 0.150 |
| rs1799750 | Collagen degradation | Andrea V 2015 | 25599395 | 0.375 | 0.339 | 0.442 | 0.520 | 0.377 | 0.345 | 0.498 | 0.413 |
| rs964184 | Vitamin E | Jacqueline M 2014 | 24623848 | 0.763 | 0.760 | 0.777 | 0.777 | 0.720 | 0.761 | 0.848 | 0.765 |
| rs7129781 | Vitamin D concentration | Sinnott-Armstrong N 2021 | 33462484 | 0.183 | 0.151 | 0.127 | 0.200 | 0.060 | 0.175 | 0.075 | 0.082 |
| rs12794714 | Vitamin D concentration | Revez JA 2020 | 32242144 | 0.372 | 0.448 | 0.352 | 0.110 | 0.528 | 0.359 | 0.463 | 0.447 |
| rs10741657 | Vitamin D concentration | Jiang X 2018 | 29343764 | 0.720 | 0.734 | 0.701 | 0.784 | 0.732 | 0.681 | 0.633 | 0.641 |
| rs1060573 | Acne | Anna H 2021 | 33849530 | 0.315 | 0.307 | 0.599 | 0.674 | 0.607 | 0.288 | 0.681 | 0.698 |
| rs526934 | Vitamin B12 concentration | John W 2015 | 25948668 | 0.823 | 0.859 | 0.799 | 0.889 | 0.758 | 0.831 | 0.720 | 0.754 |
| rs174547 | Omega concentration | Guan W 2014 | 24823311 | 0.821 | 0.844 | 0.296 | 0.021 | 0.583 | 0.580 | 0.346 | 0.143 |
| rs174576 | Omega concentration | Manja M 2020 | 33011673 | 0.821 | 0.844 | 0.366 | 0.249 | 0.607 | 0.581 | 0.355 | 0.146 |
| rs174583 | Omega concentration | Leila M 2018 | 29937909 | 0.820 | 0.844 | 0.368 | 0.242 | 0.608 | 0.584 | 0.359 | 0.158 |
| rs478304 | Acne | Alexander A 2014 | 24927181 | 0.462 | 0.505 | 0.523 | 0.591 | 0.681 | 0.527 | 0.543 | 0.269 |
| rs1695 | Vitamin E concentration | Anna E 2012 | 22572643 | 0.220 | 0.240 | 0.362 | 0.478 | 0.484 | 0.179 | 0.340 | 0.294 |
| rs4944062 | Vitamin D concentration | Jiang X 2018 | 29343764 | 0.289 | 0.271 | 0.404 | 0.333 | 0.465 | 0.385 | 0.706 | 0.158 |
| rs1042602 | Freckle | Patrick S 2007 | 17952075 | 0.004 | 0.000 | 0.128 | 0.013 | 0.247 | 0.002 | 0.375 | 0.067 |
| rs11057830 | Vitamin E concentration | Jacqueline M 2014 | 24623848 | 0.051 | 0.052 | 0.142 | 0.198 | 0.118 | 0.106 | 0.167 | 0.088 |
| rs62034322 | Stretch mark | Joyce YT 2013 | 23633020 | 0.039 | 0.047 | 0.238 | 0.219 | 0.394 | 0.087 | 0.330 | 0.191 |
| rs9923231 | Vitamin K concentration | Takeuchi F 2009 | 19300499 | 0.850 | 0.844 | 0.344 | 0.058 | 0.408 | 0.885 | 0.386 | 0.143 |
| rs11645428 | Vitamin A concentration | Beatrix F 2014 | 24586510 | 0.010 | 0.021 | 0.155 | 0.073 | 0.215 | 0.008 | 0.364 | 0.150 |
| rs6564851 | Vitamin A concentration | Xiao C 2019 | 30896431 | 0.825 | 0.792 | 0.518 | 0.349 | 0.532 | 0.808 | 0.492 | 0.502 |
| rs12934922 | Vitamin A concentration | W C Leung 2009 | 19103647 | 0.161 | 0.156 | 0.230 | 0.091 | 0.319 | 0.126 | 0.448 | 0.238 |
| rs7501331 | Vitamin A concentration | W C Leung 2009 | 19103647 | 0.203 | 0.193 | 0.149 | 0.008 | 0.169 | 0.191 | 0.232 | 0.211 |
| rs369230 | Tanning response | Alessia V 2018 | 29739929 | 0.482 | 0.479 | 0.623 | 0.463 | 0.722 | 0.473 | 0.705 | 0.838 |
| rs154659 | Tanning response | Hongmei N 2010 | 19340012 | 0.493 | 0.469 | 0.404 | 0.582 | 0.403 | 0.397 | 0.250 | 0.308 |
| rs2228479 | Freckle | Kyoko Y 2012 | 22854540 | 0.361 | 0.443 | 0.077 | 0.004 | 0.027 | 0.298 | 0.066 | 0.021 |
| rs1805007 | Freckle | Patrick S 2007 | 17952075 | 0.000 | 0.000 | 0.019 | 0.003 | 0.017 | 0.001 | 0.073 | 0.004 |
| rs885479 | UV protection | Terence H 2005 | 15963603 | 0.585 | 0.516 | 0.183 | 0.005 | 0.308 | 0.611 | 0.072 | 0.044 |
| rs11648785 | Tanning response | Nicholas E 2010 | 20585627 | 0.177 | 0.161 | 0.266 | 0.137 | 0.296 | 0.155 | 0.344 | 0.458 |
| rs2108622 | Vitamin E concentration | Maria L 2016 | 25370453 | 0.203 | 0.219 | 0.232 | 0.078 | 0.247 | 0.213 | 0.277 | 0.418 |
| rs1047781 | Vitamin B12 concentration | Keith L 2014 | 22367966 | 0.544 | 0.521 | 0.083 | 0.000 | 0.001 | 0.438 | 0.000 | 0.013 |
| rs601338 | Vitamin B12 concentration | Suraj S 2017 | 28334792 | 0.004 | 0.005 | 0.334 | 0.497 | 0.338 | 0.003 | 0.452 | 0.286 |
| rs602662 | Vitamin B12 concentration | Aditi H 2009 | 18776911 | 0.004 | 0.005 | 0.342 | 0.499 | 0.350 | 0.003 | 0.479 | 0.286 |
| rs1801131 | Vitamin B9 concentration | Shuaili Xu 2020 | 32340630 | 0.282 | 0.266 | 0.244 | 0.149 | 0.148 | 0.215 | 0.306 | 0.423 |
| rs1801133 | Vitamin B2 concentration | Carlos JGM 2014 | 25322900 | 0.173 | 0.182 | 0.246 | 0.088 | 0.477 | 0.287 | 0.367 | 0.126 |
| rs7531806 | Acne | Anna H 2021 | 33849530 | 0.483 | 0.505 | 0.569 | 0.775 | 0.452 | 0.429 | 0.563 | 0.502 |
| rs10798036 | Stretch mark | Joyce YT 2013 | 23633020 | 0.606 | 0.589 | 0.699 | 0.973 | 0.677 | 0.600 | 0.519 | 0.597 |
| rs1800896 | Vitamin E concentration | Anna E 2012 | 22572643 | 0.066 | 0.068 | 0.282 | 0.319 | 0.309 | 0.056 | 0.453 | 0.243 |
| rs4654748 | Vitamin B6 concentration | Toshiko T 2009 | 19303062 | 0.623 | 0.635 | 0.370 | 0.063 | 0.438 | 0.585 | 0.467 | 0.458 |
| rs1697421 | Vitamin B6 concentration | Keene KL 2014 | 25147783 | 0.572 | 0.542 | 0.371 | 0.071 | 0.442 | 0.523 | 0.498 | 0.476 |
| rs1780316 | Vitamin B6 concentration | Rhee EP 2013 | 23823483 | 0.985 | 0.979 | 0.929 | 0.854 | 0.956 | 0.985 | 0.933 | 0.958 |
| rs1256335 | Vitamin B6 concentration | Aditi H 2009 | 19744961 | 0.972 | 0.969 | 0.830 | 0.773 | 0.863 | 0.981 | 0.776 | 0.799 |
| rs1159268 | Acne | Anna H 2021 | 33849530 | 0.516 | 0.495 | 0.340 | 0.323 | 0.287 | 0.488 | 0.317 | 0.289 |
| rs4911414 | Freckle | Sulem P 2008 | 18488028 | 0.828 | 0.828 | 0.797 | 0.881 | 0.661 | 0.824 | 0.705 | 0.851 |
| rs1015362 | Freckle | Sulem P 2008 | 18488028 | 0.172 | 0.172 | 0.410 | 0.825 | 0.417 | 0.179 | 0.275 | 0.156 |
| rs3918242 | Elasticity | Naval J 2014 | 25061327 | 0.145 | 0.115 | 0.150 | 0.110 | 0.080 | 0.156 | 0.168 | 0.242 |
| rs6133175 | Vitamin C concentration | Eric J. D 2013 | 23737080 | 0.609 | 0.594 | 0.320 | 0.038 | 0.357 | 0.652 | 0.362 | 0.342 |
| rs6053005 | Vitamin C concentration | Eric J. D 2013 | 23737080 | 0.459 | 0.458 | 0.694 | 0.964 | 0.651 | 0.451 | 0.545 | 0.721 |
| rs6009527 | Omega concentration | Dorajoo R 2015 | 26584805 | 0.342 | 0.365 | 0.349 | 0.326 | 0.229 | 0.408 | 0.336 | 0.438 |
| rs315934 | Inflammatory Cytokine | Oudot T 2009 | 19554025 | 0.023 | 0.016 | 0.080 | 0.078 | 0.093 | 0.021 | 0.172 | 0.032 |
| rs6721961 | Antioxidation response | YaXing G 2016 | 26887053 | 0.781 | 0.844 | 0.857 | 0.943 | 0.810 | 0.750 | 0.879 | 0.849 |
| rs6706649 | Antioxidation response | Paludo 2014 | 26887053 | 0.024 | 0.036 | 0.061 | 0.009 | 0.067 | 0.063 | 0.109 | 0.080 |
| rs35652124 | Antioxidation response | Jolien 2014 | 26887053 | 0.569 | 0.583 | 0.370 | 0.151 | 0.422 | 0.546 | 0.304 | 0.553 |
| rs3910516 | Stretch mark | Joyce YT 2013 | 23633020 | 0.892 | 0.870 | 0.792 | 0.903 | 0.782 | 0.926 | 0.765 | 0.533 |
| rs12611820 | Vitamin B9 concentration | Keene KL 2014 | 25147783 | 0.455 | 0.464 | 0.393 | 0.633 | 0.212 | 0.497 | 0.194 | 0.291 |
| rs7594220 | Stretch mark | Joyce YT 2013 | 23633020 | 0.948 | 0.906 | 0.848 | 0.782 | 0.858 | 0.916 | 0.836 | 0.882 |
| rs699664 | Vitamin K concentration | Hiroyuki K 2007 | 17029979 | 0.299 | 0.359 | 0.382 | 0.661 | 0.266 | 0.312 | 0.359 | 0.153 |
| rs322458 | Skin aging | Sigrid L 2013 | 23223146 | 0.188 | 0.234 | 0.335 | 0.461 | 0.346 | 0.172 | 0.363 | 0.266 |
| rs322458 | Skin aging | Sigrid L 2013 | 23223146 | 0.188 | 0.234 | 0.335 | 0.461 | 0.346 | 0.172 | 0.363 | 0.266 |
| rs1050450 | Antioxidation response | Chunyan H 2011 | 20727719 | 0.051 | 0.052 | 0.220 | 0.265 | 0.187 | 0.069 | 0.336 | 0.205 |
| rs2282679 | Vitamin D concentration | Yun Z 2018 | 29409465 | 0.202 | 0.208 | 0.198 | 0.044 | 0.206 | 0.260 | 0.256 | 0.296 |
| rs2299007 | Moisturizing | Mariana L 2020 | 32796837 | 0.450 | 0.354 | 0.209 | 0.046 | 0.278 | 0.503 | 0.157 | 0.161 |
| rs11740584 | Moisturizing | Mariana L 2020 | 32796837 | 0.150 | 0.188 | 0.477 | 0.370 | 0.506 | 0.163 | 0.719 | 0.662 |
| rs11950646 | Vitamin C concentration | Eric J. D 2013 | 23737080 | 0.280 | 0.234 | 0.375 | 0.048 | 0.600 | 0.279 | 0.626 | 0.507 |
| rs35391 | Tanning response | Zhang M 2013 | 23548203 | 0.374 | 0.396 | 0.576 | 0.566 | 0.563 | 0.404 | 0.963 | 0.360 |
| rs629725 | Acne | Anna H 2021 | 33849530 | 0.695 | 0.682 | 0.762 | 0.934 | 0.689 | 0.731 | 0.684 | 0.676 |
| rs1264701 | Skin sensitivity | Berran Y 2016 | 27258892 | 0.350 | 0.333 | 0.178 | 0.021 | 0.157 | 0.383 | 0.225 | 0.178 |
| rs1573298 | Skin sensitivity | Berran Y 2016 | 27258892 | 0.181 | 0.156 | 0.235 | 0.135 | 0.341 | 0.257 | 0.261 | 0.247 |
| rs1557608 | Skin sensitivity | Berran Y 2016 | 27258892 | 0.183 | 0.156 | 0.274 | 0.245 | 0.363 | 0.257 | 0.296 | 0.235 |
| rs8512 | Skin sensitivity | Berran Y 2016 | 27258892 | 0.033 | 0.026 | 0.115 | 0.007 | 0.155 | 0.065 | 0.180 | 0.222 |
| rs1800629 | Inflammatory Cytokine | Anna H 2021 | 33849530 | 0.070 | 0.089 | 0.093 | 0.119 | 0.077 | 0.056 | 0.142 | 0.052 |
| rs184003 | Glycation | Marine SD 2019 | 30863465 | 0.195 | 0.188 | 0.154 | 0.194 | 0.103 | 0.145 | 0.081 | 0.221 |
| rs2070600 | Glycation | Marine SD 2019 | 30863465 | 0.173 | 0.141 | 0.068 | 0.004 | 0.012 | 0.229 | 0.051 | 0.072 |
| rs1800624 | Glycation | Marine SD 2019 | 30863465 | 0.100 | 0.130 | 0.147 | 0.010 | 0.285 | 0.136 | 0.242 | 0.150 |
| rs1800625 | Glycation | Marine SD 2019 | 30863465 | 0.125 | 0.115 | 0.134 | 0.139 | 0.077 | 0.089 | 0.153 | 0.195 |
| rs406477 | Skin sensitivity | Ferreira MA 2017 | 29083406 | 0.142 | 0.161 | 0.282 | 0.552 | 0.272 | 0.124 | 0.193 | 0.139 |
| rs1130534 | Glycation | Ying X 2020 | 31993713 | 0.225 | 0.234 | 0.236 | 0.351 | 0.163 | 0.232 | 0.128 | 0.242 |
| rs1049346 | Glycation | Ying X 2020 | 31993713 | 0.612 | 0.547 | 0.568 | 0.705 | 0.473 | 0.586 | 0.498 | 0.498 |
| rs12203592 | Freckle | Eriksson N 2010 | 20585627 | 0.001 | 0.000 | 0.038 | 0.007 | 0.068 | 0.000 | 0.121 | 0.007 |
| rs1540771 | Freckle | Patrick S 2007 | 17952075 | 0.291 | 0.323 | 0.321 | 0.109 | 0.485 | 0.282 | 0.513 | 0.336 |
| rs7765892 | Tanning response | Visconti A 2018 | 29739929 | 0.762 | 0.813 | 0.562 | 0.377 | 0.634 | 0.796 | 0.592 | 0.519 |
| rs2066853 | Skin aging | Wenshan G 2016 | 28057405 | 0.331 | 0.292 | 0.272 | 0.460 | 0.174 | 0.374 | 0.105 | 0.146 |
| rs1800796 | Inflammatory Cytokine | Anna H 2021 | 33849530 | 0.763 | 0.828 | 0.301 | 0.101 | 0.300 | 0.794 | 0.049 | 0.384 |
| rs1800795 | Elasticity | Naval J 2014 | 25061327 | 0.996 | 1.000 | 0.857 | 0.984 | 0.818 | 0.999 | 0.575 | 0.858 |
| rs7787362 | Stretch mark | Joyce YT 2013 | 23633020 | 0.195 | 0.214 | 0.294 | 0.096 | 0.355 | 0.231 | 0.550 | 0.330 |
| rs11979919 | Skin aging | Wenshan G 2016 | 28057405 | 0.118 | 0.125 | 0.185 | 0.320 | 0.126 | 0.138 | 0.085 | 0.185 |
| rs4133274 | Acne | Mingfeng Z 2014 | 24114350 | 0.243 | 0.260 | 0.149 | 0.118 | 0.056 | 0.223 | 0.062 | 0.292 |

**Table S2.** Correlation p-value of proportion of each status of Vietnamese microarray dataset compared with other populations.

|  | Worse-than-average | Better-than-average | Average |
| --- | --- | --- | --- |
| VN vs. EAS | 1.256298e-10 | 3.201401e-13 | 2.184168e-14 |
| VN vs. AFR | 7.040610e-02 | 4.330830e-02 | 2.880256e-02 |
| VN vs. AMR | 2.444896e-03 | 7.607991e-04 | 9.726836e-03 |
| VN vs. EUR | 3.197021e-02 | 9.902911e-04 | 4.175825e-03 |
| VN vs. SAS | 4.232898e-03 | 3.841360e-03 | 1.104794e-03 |

**Table S3. Correlation among genetic risk of skin traits and daily habits.**

| **Pairwise** | **p** | **PearsonCC** | **Pairwise** | **p** | **PearsonCC** |
| --- | --- | --- | --- | --- | --- |
| Collagen degradation:Elasticity | 1,48e-23 | 0,840715 | alcohol_used:intensive_care | 0,017724 | -0,2582 |
| Vitamin B2:Vitamin B9 | 3,61e-12 | 0,668737 | Skin aging:Skin sensitivity | 0,018 | -0,2576 |
| gender:skincare | 5,03e-09 | 0,585214 | age:skincare | 0,018868 | 0,255757 |
| gender:alcohol_used | 6,18e-07 | -0,51265 | Vitamin A:stay_up_late | 0,018914 | 0,255663 |
| Skin aging:Wrinkle | 1,5e-05 | 0,453205 | Tanning response:gender | 0,019154 | -0,25517 |
| intensive_care:skincare | 0,000255 | 0,38904 | Vitamin C:sweet | 0,019528 | -0,25441 |
| Collagen degradation:Moisturizing | 0,000805 | 0,358747 | Vitamin A:age | 0,019655 | -0,25415 |
| Vitamin A:sweet | 0,001055 | 0,351199 | Vitamin K:skincare | 0,020195 | 0,25308 |
| Antioxidation response:exercise | 0,001075 | -0,35066 | Moisturizing:Vitamin A | 0,020373 | 0,252732 |
| Elasticity:Moisturizing | 0,0011 | 0,349997 | Vitamin B6:medicine_used | 0,020506 | -0,25247 |
| Collagen degradation:Skin aging | 0,001378 | 0,343522 | Vitamin D:alcohol_used | 0,020785 | -0,25194 |
| Tanning response:Vitamin B9 | 0,002648 | -0,32394 | exercise:stay_up_late | 0,02096 | -0,2516 |
| enviroment:intensive_care | 0,003599 | 0,31427 | age:exercise | 0,021511 | 0,250568 |
| gender:intensive_care | 0,004891 | 0,30429 | exercise:water_liters | 0,023707 | 0,246657 |
| age:gender | 0,006173 | 0,296482 | Stretch mark:Tanning response | 0,023786 | -0,24652 |
| gender:satisfaction | 0,00921 | -0,28256 | Acne:satisfaction | 0,025322 | -0,24397 |
| Tanning response:alcohol_used | 0,010686 | 0,277206 | Vitamin E:alcohol_used | 0,025645 | -0,24345 |
| Elasticity:Sagging eyelid | 0,013759 | 0,267872 | gender:exercise | 0,026145 | -0,24266 |
| Elasticity:Skin aging | 0,016644 | 0,260631 | age:medicine_used | 0,027216 | 0,241004 |
| Wrinkle:alcohol_used | 0,017581 | -0,25851 | Antioxidation response:Vitamin B6 | 0,028232 | -0,23948 |
